# Multi-omics factor analysis identifies the Tensin 1-Fermitin family homologue 2–Fibronectin 1–Integrin signaling axis as a prognostic determinant in colorectal cancer

**DOI:** 10.1186/s43556-025-00386-0

**Published:** 2025-12-17

**Authors:** Tianwei Chen, Yebin Yang, Jing Shi, Fanhe Dong, Lesi Xie, Yuqiang Shan, Xiang Wang

**Affiliations:** 1 Zhejiang Key Laboratory of Zero Magnetic Medicine, School of Medicine, Affiliated Hangzhou First People’s Hospital, Westlake University, Hangzhou, China; 2 Department of Gastrointestinal Surgery, School of Medicine, Affiliated Hangzhou First People’s Hospital, Westlake University, Hangzhou, China; 3 Department of General Surgery, Zhejiang Hospital, Hangzhou, Zhejiang Province China; 4 Department of Pathology, School of Medicine, Affiliated Hangzhou First People’s Hospital, Westlake University, Hangzhou, China

**Keywords:** Colorectal cancer, Multi-omics factor analysis, Myofibroblasts, Extracellular matrix, TNS1, FERMT2

## Abstract

Colorectal cancer (CRC) remains a leading cause of cancer-related mortality worldwide, with its heterogeneous molecular profiles complicating prognosis prediction. Multi-omics profiling of clinical CRC specimens is particularly valuable for uncovering prognostic variables; however, such integrated studies remain scarce. Here, we applied the Multi-Omics Factor Analysis v2 framework to multi-omics data (mutations, miRNA, RNA, proteomics, phospho-proteomics) from the CPTAC-2 cohort and constructed a comprehensive CRC model. This approach identified a survival-associated latent variable, which we term the CRC Prognostic Latent Factor (CPLF). The prognostic relevance of CPLF was then rigorously validated across three independent multi-omics cohorts, encompassing 579 patients with CRC. CPLF primarily reflects extracellular matrix deposition in the tumour microenvironment, with Tensin 1 (TNS1) and Fermitin family homologue 2 (FERMT2) as the highest-weighted features. Single-cell and spatial transcriptomic analyses, supplemented by immunohistochemistry, localized CPLF-associated gene expression predominantly to myofibroblasts. Functionally, knockdown of Tensin 1 or FERMT2 in fibroblasts attenuated tumour growth in vivo. Consistently, these top-weighted components of CPLF upregulated fibronectin 1 (FN1) expression in myofibroblasts, thereby activating integrin signaling in cancer cells and enhancing tumour progression. Altogether, our findings unveil CPLF as a data-inherent multi-omics prognostic factor and establish the CPLF/FN1/integrin axis as a key pathway mediating myofibroblast–cancer cell crosstalk in CRC progression.

**Supplementary Information:**

The online version contains supplementary material available at 10.1186/s43556-025-00386-0.

## Introduction

Multi-omics profiling of colorectal cancer (CRC) specimens comprehensively characterizes the molecular landscape of tumours, establishing integrative approaches as powerful tools for dissecting cancer complexity [1]. While each omics layer provides a valuable perspective, no single modality can reveal the systems-level logic underlying coordinated tumour progression. Integration of multiple layers enables the mapping of synergistic driver networks across omics, identifying key hubs driving tumour progression and deciphering complementary mechanisms that are otherwise underscored by single omics. Among these layers, proteomics directly reflects functional genomics, while phosphorylation is one of the most central and rapid post-translational modifications that directly regulate protein function and activity. Crucially, phospho-proteomics captures the real-time dynamics of intracellular signaling pathways, which often operate through on/off phosphorylation events, providing insights that are inaccessible through other layers.

Conventional CRC multi-omics studies have typically analysed individual, isolated layers or employed only marginal association techniques, thereby overlooking the synergistic regulatory mechanisms embedded within interconnected molecular hierarchies. Kernel- or graph-based clustering fails to elucidate the molecular determinants governing graph structures, whereas factor analysis directly reveals latent data features with inherent interpretability. The Multi-Omics Factor Analysis v2 (MOFA +) framework addresses key challenges in multimodal data by decomposing the global covariance matrix into latent factors that represent shared biological variation across omics layers [2]. Specifically, MOFA + offers three distinct advantages: it explicitly handles the common multi-omics challenge of "missing views" through its probabilistic Bayesian framework, which learns a coherent model from all available data without imputation, thereby increasing statistical power and avoiding imputation-related biases; its flexibility accommodates diverse data types across any number of layers; it provides interpretable factors that represent latent sources of variation and enable the identification of key biological drivers. Compared with earlier tools such as iCluster [3], MOFA + demonstrates superior performance when integrating more than three data types. However, the presence of hidden sources of variance in multi-omics prognosis data from patients with CRC has not been systematically investigated.

Myofibroblasts, first described by Gabbiani et al. in 1971 as contractile alpha-smooth muscle actin (α-SMA)-positive stromal cells with hybrid smooth muscle-fibroblast features [4], exert approximately twice the contractile force of fibroblasts through stress-fibre assembly [4]. These cells dynamically remodel the extracellular matrix (ECM) via collagen I/III, fibronectin, and hyaluronan deposition [5]. While initially characterized in wound healing, they are now recognized as key mediators of pathological fibrosis and tumour progression [5]. In CRC, tumour-associated myofibroblasts display expanded functional plasticity, engaging in immunomodulation [6], metastatic niche formation [7], and ECM-driven therapeutic resistance [8]. Despite advances in single-cell transcriptomic classification [5], two critical gaps persist: (1) the identity of key regulators mediating tumour-promoting functions in myofibroblasts; (2) the mechanisms underlying cancer progression triggered by myofibroblast activation. Tensin 1 (TNS1), a component of the focal adhesion complex that mediates extracellular matrix (ECM) sensing and myofibroblast differentiation, has an unclear role in CRC. Similarly, the function of Fermitin family homologue 2 (FERMT2), a scaffold protein involved in ECM sensing, intracellular signal transduction, and cell spreading, remains unknown.

In this study, we hypothesized that tumour pathogenesis is not driven by alterations within a single molecular layer but from dynamic interactions across genomic, epigenomic, transcriptomic, proteomic, and phospho-proteomic layers. Leveraging MOFA +, we constructed a low-dimensional integrative model of genomic, transcriptomic, and proteomic data from 96 CRC specimens, through which we identified a novel prognostic determinant termed CRC Prognostic Latent Factor (CPLF). Its clinical relevance was rigorously validated in three independent multi-omics cohorts encompassing 579 patients with CRC. Multivariable Cox analysis confirmed its prognostic value as independent of conventional Tumour, Node, Metastasis (TNM) staging—highlighting its potential as a complementary risk-stratification biomarker. We further demonstrated that genes loading strongly onto CPLF show enriched expression in myofibroblasts, and mechanistically unveiled that its key molecular components, FERMT2 and TNS1, promote tumour progression by enhancing fibronectin 1 (FN1) abundance and activating the integrin-linked kinase/focal adhesion kinase (ILK/FAK) signaling axis. By systematically dissecting the cellular origin, functional impact, and mechanistic basis of a myofibroblast-driven prognostic signature, this work provides actionable insights into CRC biology and establishes a translatable framework that bridges multi-omics discovery with experimental validation. Thus, our study directly addresses several unresolved clinical questions by uncovering a major latent variable affecting CRC prognosis, delineating the pro-tumorigenic role of myofibroblasts, and elucidating the functional contribution of FERMT2 and TNS1 to disease progression. Our findings may pave the way for novel stroma-targeted therapeutic strategies.

## Results

### Integrative multi-omics analysis of CRC reveals MOFA-derived latent factors

**Fig. 1:**
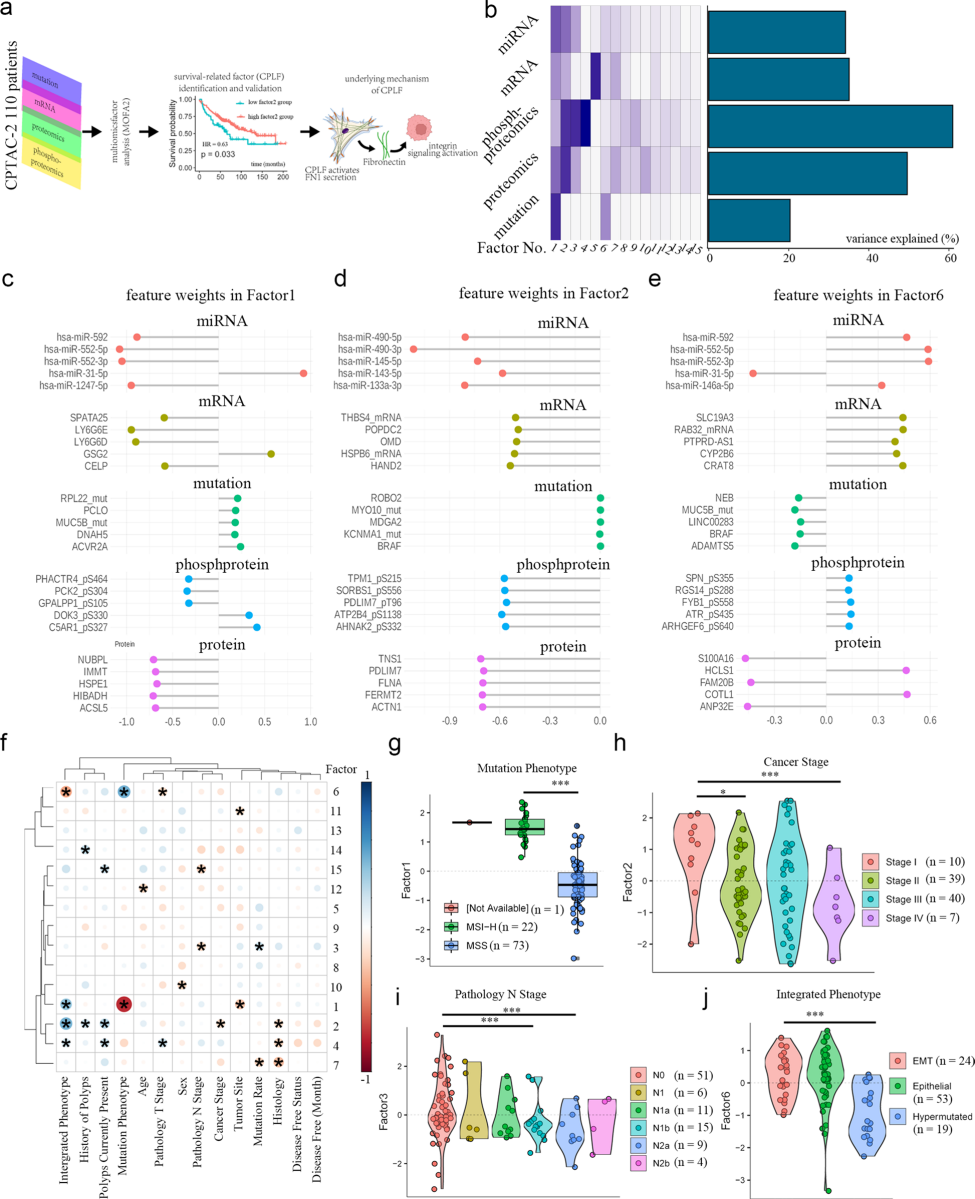
MOFA + decomposes multi-omic data of CRC. **a** schematic flowchart of this study. **b** Bar plot showing variance explained by different factors. **c**-**e** Lollipop chart indicating feature weights of Factor 1(**c**), Factor 2 (**d**) and Factor 6 (**e**). **f** Coloured plot showing the correlation between clinicopathological data and factor scores. *, *P* < 0.05. **g** Boxplot demonstrating scores of Factor 1 in different microsatellite status group CRC patients. ***, *P* < 0.001. **h**-**j** Violin plot showing factor 2 score in CRC patients of different cancer stage (**h**), factor 3 score in CRC patients of different pathology N stage (**i**), factor 6 score in CRC patients of different Integrated phenotype (**j**).*, *P* < 0.05; ***, *P* < 0.001

We applied the MOFA + framework to identify hidden variables underpinning patient heterogeneity in CRC using multi-omics data. Somatic mutations, transcriptomics, proteomics, and phospho-proteomics data from 110 patients in the CPTAC-2 cohort were integrated [9], followed by functional validation (Fig. 1a). The clinical characteristics of the cohort are provided in Table S1. After normalizing and filtering omics layers (Materials and methods), the model converged on 15 latent factors, collectively explaining 40.5% of the total variance (Fig. 1b). Notably, these MOFA-derived factors (MDFs) captured 60% of the variance in phospho-proteomics data but only 20% of the variance in the somatic mutation profiles.

To gain insight into the gene loadings of each MDF, genes were ranked by feature weights. The top-weighted genes included hsa-miR-31-5p and *LY6G6E* in MDF1 (Fig. 1c), hsa-miR-490-3p and *TNS1* in MDF2 (Fig. 1d), hsa-miR-552-3p and *COTL1* in MDF6 (Fig. 1e). Correlation analysis between factor scores and clinicopathological parameters demonstrated that MDF1 was strongly associated with microsatellite instability status (*r* = − 0.75, *P* < 0.001, FDR < 0.001), while MDF2 was associated with TNM stage (*r* = − 0.21, *P* = 0.036, FDR = 0.329) (Fig. 1f). High MDF1 scores were associated with microsatellite instability-high status (*P* < 0.001; Fig. 1g), whereas low MDF2 scores reflected a higher TNM stage (*P* < 0.01; Fig. 1h). Additionally, lower MDF3 scores were linked with more lymph node metastasis (*P* < 0.01; Fig. 1i), while MDF6 scores corresponded to the integrated phenotype (*P* < 0.01; Fig. 1j), as demonstrated by Vasaikar [9]. Together, these findings identify latent dimensional factors associated with clinicopathological outcomes based on molecular multi-omics analysis.

### Identification and cross-cohort validation of the prognostic factor CPLF

**Fig. 2:**
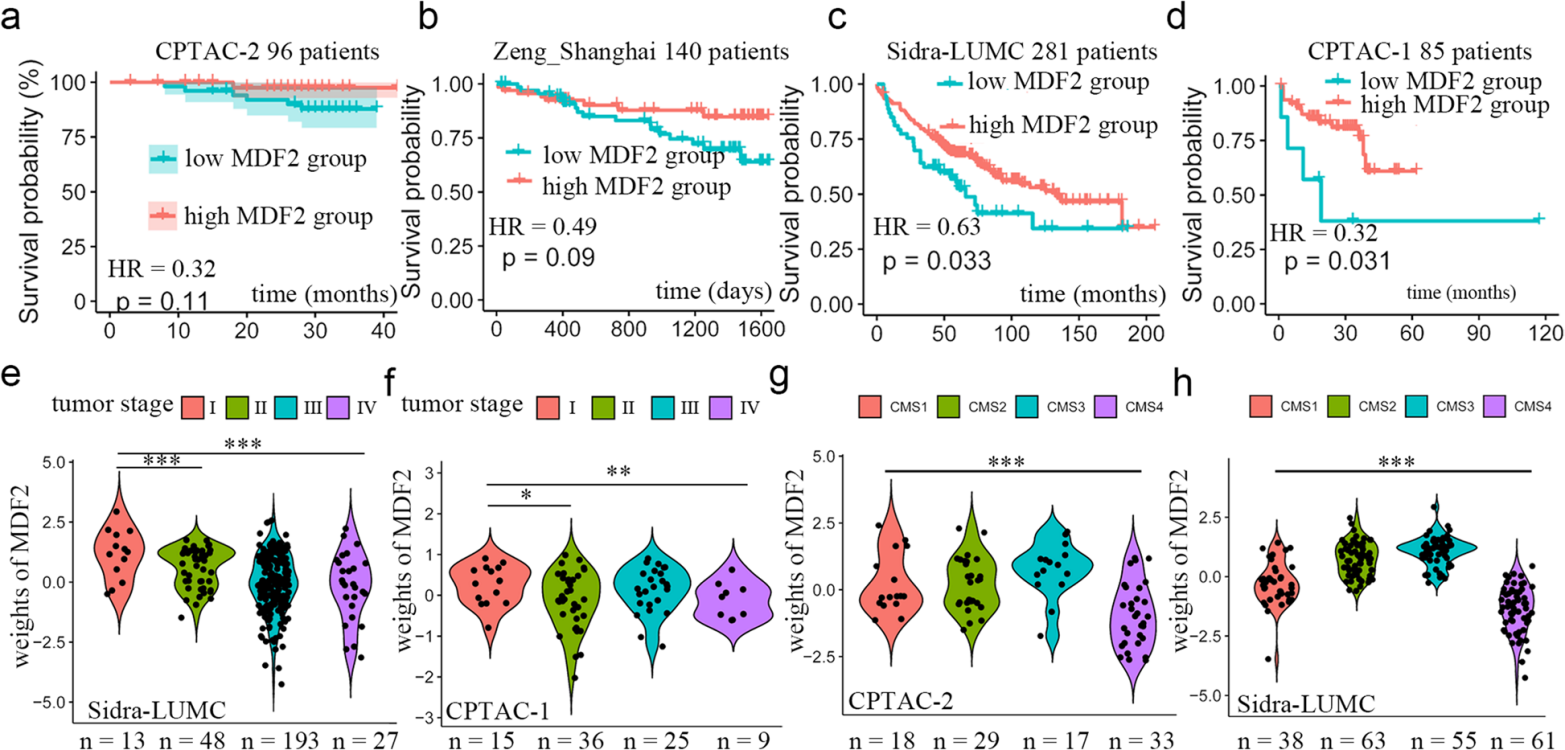
MDF2 predicts patients’ prognosis. **a**-**d** Kaplan–Meier plot showing correlation of survival status and MDF2 score in cohort CPTAC-2 (samples in low group = 51, samples in high group = 45) (**a**), Zeng_Shanghai (samples in low group = 67, samples in high group = 70) (**b**), Sidra_LUMC (samples in low group = 53, samples in high group = 228) (**c**) and CPTAC-1 (samples in low group = 12, samples in high group = 73) (**d**). **e**&**f** Violin plot showing Factor score in CRC patients of different tumor TNM stages in cohort Sidra_LUMC (**e**) and cohort CPTAC 1 (**f**). *, *P* < 0.05; ***, *P* < 0.001. **g**&**h** Violin plot showing MDF2 score in in CRC patients of different CMS subtypes in cohort CPTAC 2 (**g**) and cohort Sidra_LUMC (**h**). CMS, Consensus Molecular Subtype. *P* < 0.05; ***, *P* < 0.001, Kruskal–Wallis test

We next focused on MDF2 because of its potential prognostic value. Although low MDF2 scores showed a non-significant association with reduced overall survival (hazard ratio, HR = 0.32, *P* = 0.11; Fig. 2a) in the CPTAC-2 discovery cohort, the cohort had a median follow-up of 3 years. We therefore performed cross-cohort validation by projecting MDF2 scores onto three independent multi-omics datasets through matrix inversion of the pre-trained model's feature weights. Cox analysis revealed that MDF2 scores consistently stratified survival risk across these cohorts (total *n* = 579). Specifically, MDF2 replicated as a prognostic signature in Zeng_Shanghai [10] (*n* = 146; HR = 0.49, *P* = 0.09; Fig. 2b; detailed clinical data in Table S2), Sidra_LUMC [11] (*n* = 348; HR = 0.63, *P* = 0.033; Fig. 2c; clinical data in Table S3), and CPTAC-1 [12] (*n* = 85; HR = 0.32, *P* = 0.031; Fig. 2d; clinical data in Table S4). Moreover, low MDF2 scores correlated with advanced TNM stages in both Sidra_LUMC and CPTAC-1 (*P* < 0.01; Fig. 2e&f).

Transcriptome-wide profiling stratified CRC into four Consensus Molecular Subtypes (CMS1–4) [13], a classification framework established through integrative analysis of mRNA expression patterns across large patient cohorts. Here, our analysis revealed a significant association between low MDF2 scores and CMS4 (*P* < 0.001; Fig. 2g&h), a mesenchymal phenotype characterized by stromal infiltration and poor prognosis. Based on these findings, we identify MDF2 as a novel, data-inherent prognostic determinant in CRC.

To ascertain whether MDF2 was influenced by our initial selective filtering of mRNAs and miRNAs, we reconstructed the MOFA model without any pre-filtering, thereby employing a broader dataset for validation (Materials and methods; Fig. S1a). The resulting factors showed strong correlation with those from the original model, with 11 out of 15 factors exhibiting correlation coefficients > 0.8 (Fig. S1b). Importantly, MDF2 in the new model remained highly correlated with the original MDF2 (*r* = 0.986), retained TNS1 and FERMT2 as the top-weighted features (Fig. S1c), and preserved its prognostic value (Fig. S1d).

Next, we investigated whether the prognostic value of MDF2 was independent of other clinical covariates by performing multivariable Cox proportional hazards regression. The multivariable model did not converge for both CPTAC-2 and CPTAC-1 cohorts, likely due to limited sample sizes. For the Zeng_Shanghai cohort, RNA sequencing (RNA-seq) data were unavailable; therefore, CMS classification was omitted. Z-scores were available for 142 patients. To avoid complete separation, we excluded one patient with stage 0 and 15 with stage I disease, as no deaths occurred in these subgroups. The final model included 126 patients, with stage, sex, age, tumour site, metastasis status, and all 15 MDFs as covariates. The MDF2 score emerged as an independent, negative prognostic factor [HR (95% confidence interval, CI) = 0.2 (0.04–0.97), *P* = 0.045] (Fig. S2a). Similarly, in the Sidra_LUMC cohort, the MDF2 score remained an independent, negative prognostic factor [HR (95% CI) = 0.87 (0.78–0.98), *P* = 0.018] (Fig. S2b). These results indicate that MDF2 is an independent predictor of survival in CRC, after adjusting for other clinical covariates.

Collectively, MDF2, derived through multi-omics integration, defines a robust prognostic signature that consistently stratifies patients with CRC into distinct survival groups, with validation across three independent cohorts (*n* = 579). We thus designate MDF2 as CPLF.

### CPLF primarily reflects ECM remodelling

The MOFA + feature weights quantify the relative contribution of each molecular feature to CPLF, with Z-score standardization enabling direct cross-omics comparison of weights between heterogeneous data types. The miRNA profiling depth (982 miRNAs ranked by standard deviation; top 100 miRNAs used in model construction, potentially causing selection bias) was lower than that of other omics layers, including transcriptome (13,484 genes), proteome (8067 proteins), and phospho-proteome (31,262 sites). Therefore, the cross-omics feature weight analyses were mainly focused on the mRNA-protein-phosphoprotein triad to ensure robust statistical comparisons.

**Fig. 3:**
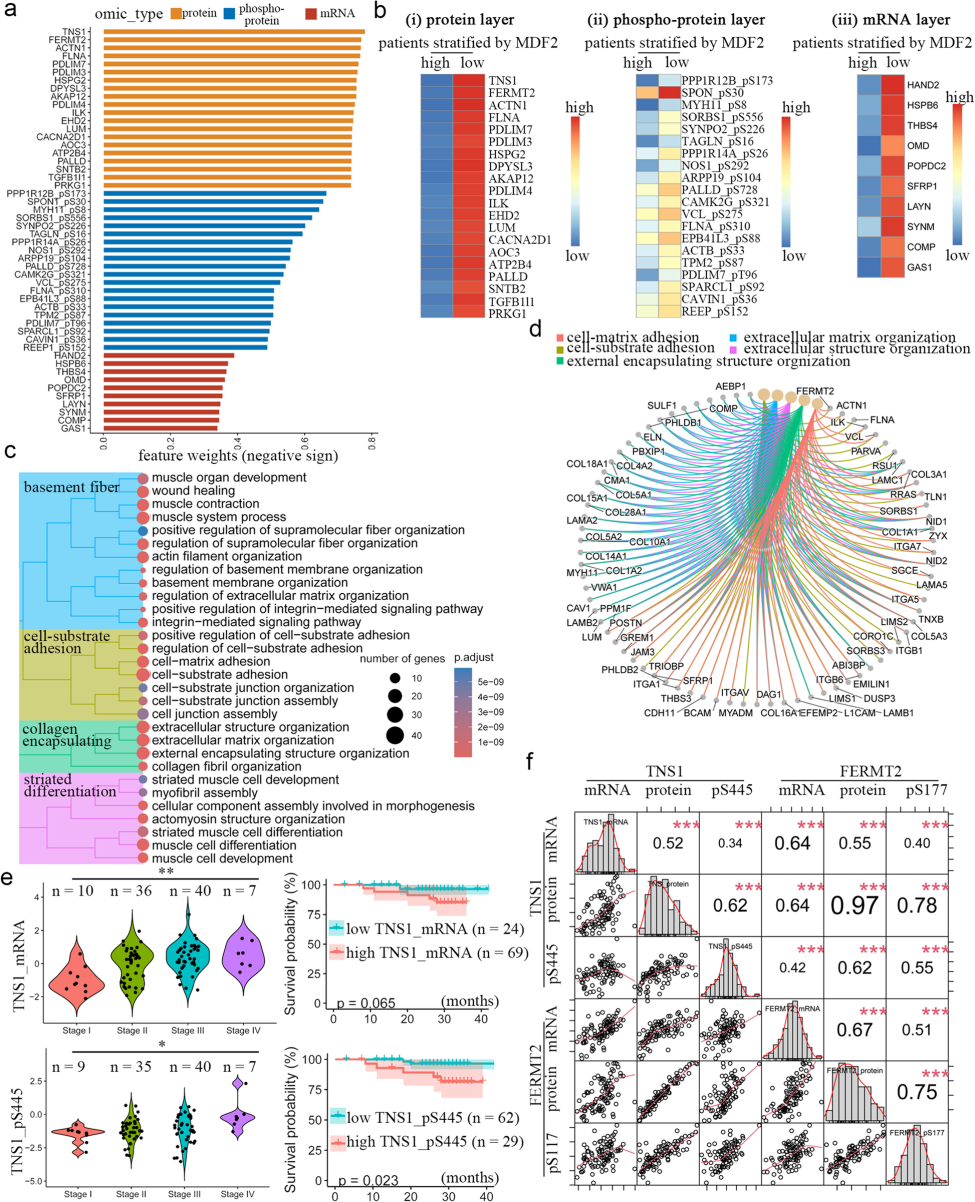
ECM remodeling enriched in CPLF. **a** Barplot showing feature weights from different omic layers. Shown are in negative sign. **b** Heatplot showing differentially expressed molecules in groups stratified by MDF2 score. **c** Hierarchy tree demonstrating enriched pathways of top 10% of feature weights in MDF2. **d** Circled pathway-gene view showing pathways and genes enriched in MDF2. **e** Violin plot and kaplan–Meier survival curve demonstrating *TNS1* mRNA levels (upper) and phosphorylated TNS1 in different TNM stage and survival status. **f** Plot showing correlation between indicated expressions different omic layers

Proteomic features exhibited markedly higher factor loadings than both phospho-proteomic sites and transcriptomic profiles in CPLF (Fig. 3a). Stratification of patients by CPLF scores revealed distinct molecular profiles through differential expression analysis. Specifically, the CPLF-low subgroup exhibited: (1) elevated protein abundance of TNS1 and FERMT2; (2) hyperphosphorylation of PPP1R12B at S173 and SPON1 at S30; (3) upregulated mRNA expression of *HAND2* and *HSPB6* (Fig. 3b).

To elucidate pathway-level implications of the top-weighted features, we first optimized proteomic factor loadings through varimax rotation—an orthogonal transformation maximizing feature variance. Next, we performed Principal Component Gene Set Enrichment [14] with 1,000 permutations. Features in the top 10% loading distribution were prioritized, revealing significant enrichment (FDR < 0.001) for pathways related to cell–matrix interactions and ECM organization (Fig. 3c&d). TNS1 emerged as the highest-weighted molecular feature (negative loading) in CPLF, with higher mRNA expression associated with advanced TNM stages (*P* = 0.004), and a non-significant trend toward worse survival outcomes (*P* = 0.065; Fig. 3e). Notably, its phosphorylation at Ser445 showed stronger clinical relevance, significantly correlating with both advanced TNM classification (*P* = 0.039) and reduced overall survival (*P* = 0.023; Fig. 3e).

Since mRNA and protein correlation only reached 0.23, as reported by Zhang [12], we speculated whether the CLPF top-weighted genes represent consistent regulation of mRNA, protein, and phospho-protein. Significantly positive correlations were observed for both TNS1 (mRNA and protein: 0.52; protein and phosphoprotein: 0.62; Fig. 3f) and FERMT2 (mRNA and protein: 0.67; protein and phosphoprotein: 0.75; Fig. 3f). The results indicate that CPLF genes were stable at the mRNA and protein level (as conceptualized by Zhang [12]), supporting the conclusion that CPLF primarily represents an ECM-related process in tumour progression.

### CPLF-associated genes are predominantly expressed by myofibroblasts

**Fig. 4:**
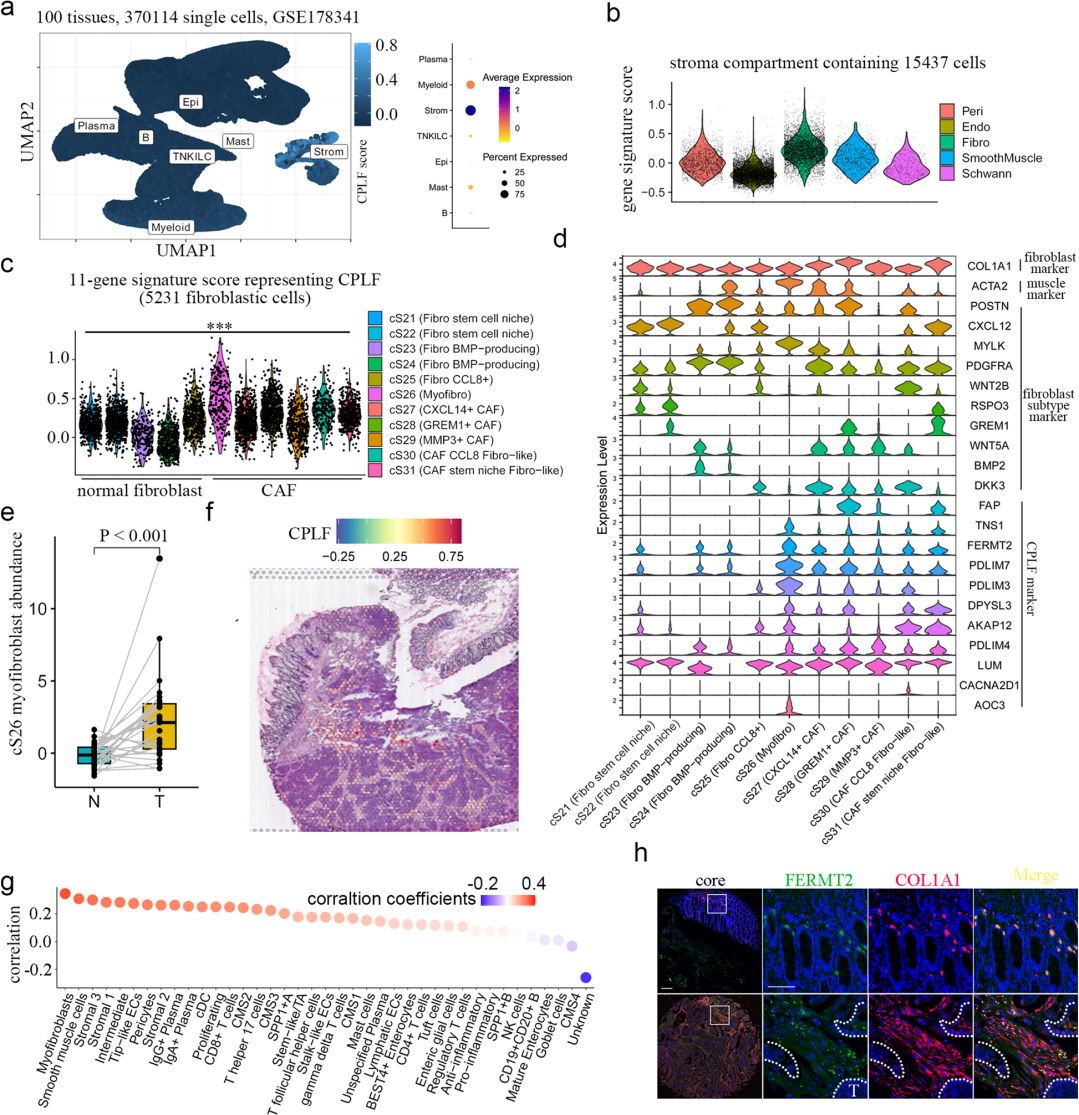
CPLF mainly expressed in myofibroblasts. **a** UMAP plot showing CPLF scores in different cancer types. **b** Violin plot showing CPLF derived 11-gene signature expression in different subtypes of stroma cells. **c** Violin plot showing CPLF derived 11-gene signature expression in different subtypes of fibroblast cells. **d** Stacked violin plot showing gene expression in different cell types. **e** Boxplot showing abundance of myofibroblasts in normal and Tumor tissues (*n* = 27). N, normal tissue; T, tumor tissues. **f** Spatial plot showing CPLF signature expression. **g** Dot plot showing the correlation coefficients between CPLF and different deconvoluted cell types. **h** Multiplex IHC staining of indicated molecules in clinical specimen

To determine the cellular origin of CPLF activity, we analysed single-cell RNA-seq (scRNA-seq) data from 371,223 cells across 100 CRC samples (GSE178341). An initial 143-gene signature was derived from CPLF feature weights (Materials and methods). Uniform Manifold Approximation and Projection mapping revealed pronounced enrichment in stromal compartments (Fig. 4a), with fibroblasts showing the highest signature scores (Fig. 4b). Since the initial scoring did not effectively discriminate myofibroblasts from stem-niche cancer-associated fibroblasts (Fig. S3a), we constructed a refined 11-gene signature based on the original feature weights (Materials and methods). This refined signature scoring successfully identified myofibroblasts as the highest-expressing cellular subset (Fig. 4c&d). Tumour tissues exhibited a fivefold increase in CPLF-positive myofibroblasts compared to normal adjacent tissue (*P* < 0.001) (Fig. 4e). Furthermore, this expression pattern could be replicated in another high-quality scRNA-seq dataset (GSE144735; Fig. S3b-e), indicating that CPLF-associated genes mainly originated from myofibroblasts in CRC.

Spatial transcriptomics analysis further validated these findings. The CPLF gene signature was significantly enriched in the stromal regions surrounding cancer cells (FDR < 0.01; Fig. 4f). To more precisely investigate the spatial relationship between CPLF and myofibroblasts, we deconvolved the spatial transcriptomics data using Spotlight (Fig. S4a). Spatial correlation analysis identified myofibroblasts as the cell type exhibiting the highest correlation with CPLF (Fig. 4g) and resolved them into two distinct spatial clusters: stromal-embedded and tumour-adjacent (Fig. S4b). Interaction profiling revealed preferential engagement with CMS2, CMS3, and intermediate cancer subtypes (Fig. S4c). Multiplex immunofluorescence co-staining of FERMT2 and the stromal marker COL1A1 (in human colorectal tissues) further supported these findings. Quantitative spatial analysis revealed distinct compartmentalization patterns: in normal colon mucosa, FERMT2 + myofibroblasts were tightly juxtaposed to the basal lamina of epithelial crypts. In contrast, tumour tissues exhibited a 4.7-fold increase in FERMT2 stromal density (*P* < 0.001), with 83% of FERMT2 signal localized to desmoplastic regions rather than tumour epithelium (Fig. 4h). This spatial redistribution suggests tumor microenvironment-driven reprogramming of myofibroblast signaling niches.

### TNS1 and FERMT2 inhibition decreases tumour growth

**Fig. 5:**
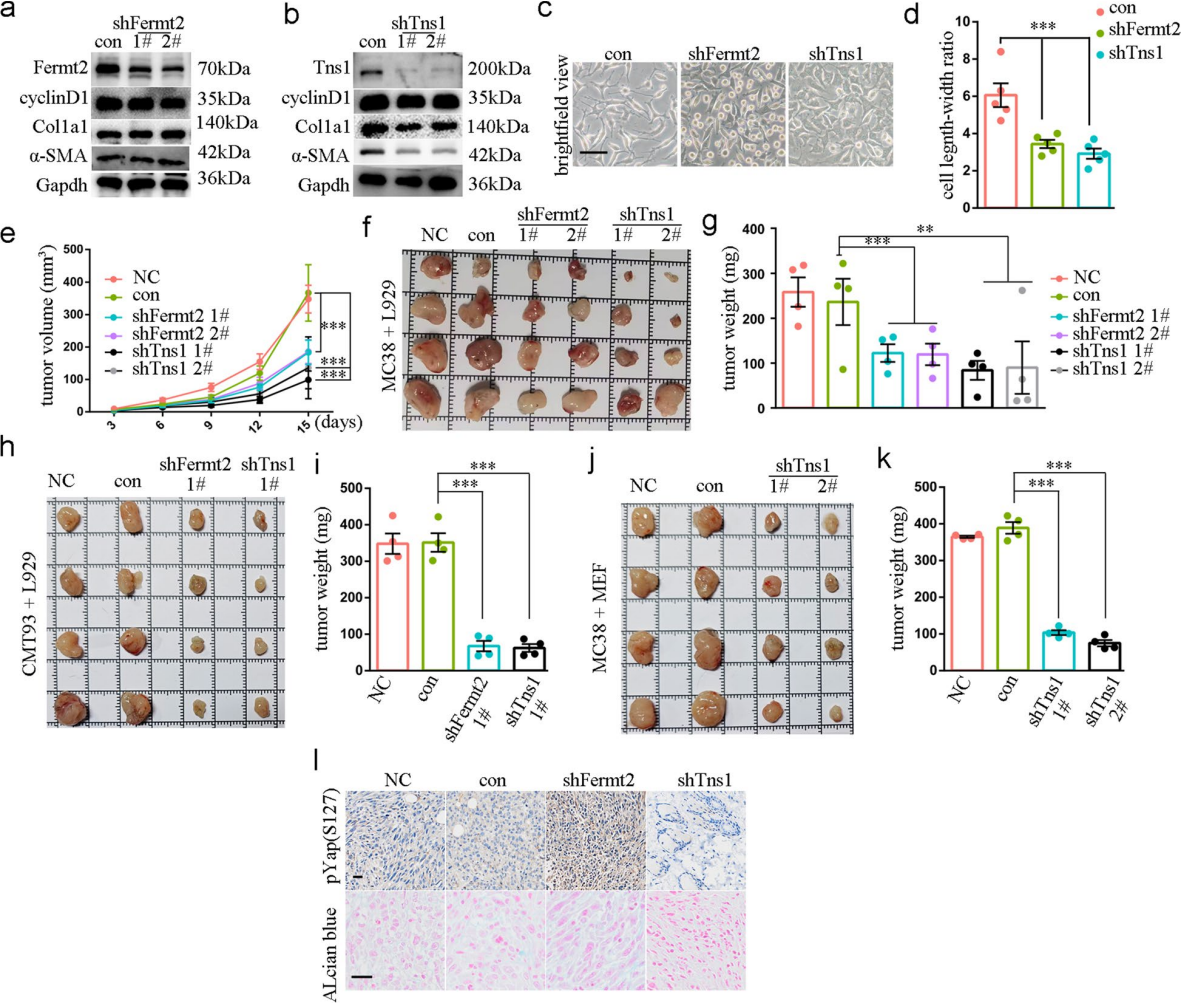
Inhibition of TNS1 and FERMT2 in myofibroblasts decreased tumor formation. **a**&**b** WB result showing the expression of indicated molecules. **c** Brightfield view showing shapes of cells. scale, 50 μm. **d** Box plot showing length–width ratio of cells, *n* = 5 for each group. ***, *P* < 0.001. **e** Lineplot showing tumor volume at different timepoints, *n* = 4 for each group. ***, *P* < 0.001. **f** Brightfield picture showing subcutaneous tumors of different groups. **g** Tumor weight of different groups, *n* = 4 for each group. **, *P* < 0.01; ***, *P* < 0.001. **h** Brightfield picture showing subcutaneous tumors of different groups. **i** Tumor weight of different groups, *n* = 4 for each group. ***, *P* < 0.001. **j** Brightfield picture showing subcutaneous tumors of different groups. **k** Tumor weight of different groups, *n* = 4 for each group. ***, *P* < 0.001. **l** Representative images showing signal intensity of indicated staining of tumors from MC38 and L929 co-culture. scale, 60 μm

TNS1 and FERMT2 are essential for focal adhesion assembly, yet their functional significance in CRC remains elusive. To validate their pro-tumorigenic role, we established stable knockdown models of murine L929 fibroblasts using lentiviral shRNA. Successful knockdown of both *Fermt2* and *Tns1*—as confirmed by western blot analysis (Fig. 5a&b, respectively)—resulted in shorter, spindle-shaped morphology (Fig. 5c), with significantly reduced length–width ratio (Fig. 5d). *Fermt2* knockdown additionally yielded more rounded cells (Fig. 5c). Neither knockdown affected L929 cell growth, as indicated by cyclin D1 expression (Fig. 5a) and Cell Counting Kit-8 assays (Fig. S5). However, consistent with prior reports from Bernau [15], *Tns1* knockdown reduced α-SMA expression (Fig. 5b).

Co-implantation of these engineered fibroblasts with MC38 colorectal carcinoma cells into syngeneic C57BL/6 mice revealed profound tumour growth suppression (Fig. 5e), and both shTns1 and shFermt2 groups exhibited significantly decreased tumour weight (Fig. 5f&g). To corroborate these findings, we performed two additional experiments. First, co-implantation of the engineered fibroblasts with another murine colon cancer cell line CMT93, which confirmed that knockdown of *Fermt2* or *Tns1* suppressed tumour growth in vivo (Fig. 5h&i). Second, to assess the broader relevance of our findings, we tested additional murine fibroblastic cell lines. However, *Fermt2* knockdown induced cell death in mouse embryonic fibroblasts (MEFs), precluding further in vivo investigation. In contrast, *Tns1* knockdown in MEFs significantly delayed MC38 tumour growth (Fig. 5j&k, Fig. S6a).

Since myofibroblasts are key producers of the ECM, which is largely composed of glycosaminoglycans and fibrous proteins [16], we first assessed glycosaminoglycan content. Alcian blue staining revealed no differences in extracellular glycosaminoglycans across experimental groups (Fig. 5l), indicating that sugars do not mediate TNS1 and FERMT2 function in fibroblasts. Fibrous protein staining results are presented in the subsequent experimental results. *Fermt2* knockdown significantly increased YAP phosphorylation at Ser127, which indicated Hippo activation through cell–cell contact (Fig. 5l). Collectively, these results demonstrate that knockdown of fibroblast-derived TNS1 and FERMT2 impedes CRC tumour growth.

### Fibronectin signaling orchestrates myofibroblast-cancer cell crosstalk

**Fig. 6:**
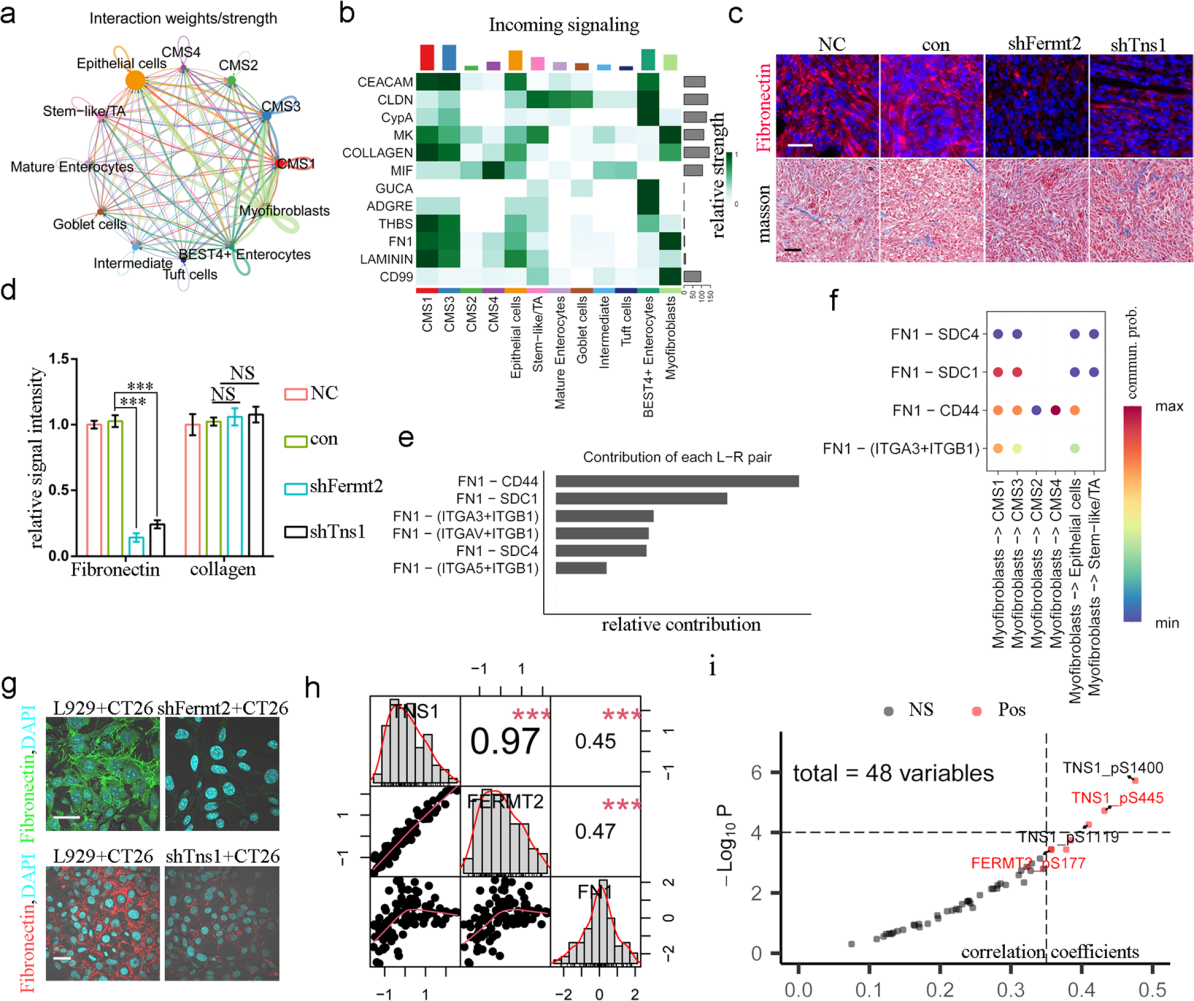
TNS1 and FERMT2 expression in myofibroblasts activated Fibronectin expression. **a** circled line plot showing crosstalk between different cell types. **b** Heatplot showing different incoming signals received by different epithelial cell types. **c** Representative images showing tumors from MC38 and L929 co-culture staining with indicated module. scale, 100 μm. **d** Barplot showing quantitative result of signal intensity in C, *n* = 3 for biological replicates. ***, *P* < 0.001; NS, not significant. **e** Barplot showing relative contribution of ligand/receptor pair to myofibroblast-epithelial crosstalk. **f** Heatplot showing communication probability of ligand/receptor pairs in different cells. **g** Representative confocal laser scanning microscope images showing the signal intensity of indicated molecules. scale, 80 μm. **h** Plot showing the correlation of protein expression of different molecules. ***, *P* < 0.001. **i** Scatterplot showing correlation of protein expression of fibronectin and different phosphorylation status of TNS1 and FERMT2

The aforementioned co-transplantation model indicated that crosstalk between myofibroblasts and cancer cells drives tumour growth. Therefore, we analysed myofibroblast–cancer cell communication using scRNA-seq data (GSE144735), which included high-quality annotations for 1226 myofibroblasts (mean features: 2551 per cell) and 6168 (mean features: 2498 per cell) cancerous cells. As stated earlier, myofibroblasts possessed the highest CPLF scores (Fig. S3d). Cellchat analysis revealed a robust communication between the two cell types, with myofibroblasts acting as dominant signal senders (Fig. 6a and Fig. S3e). The major ECM signals included COLLAGEN, FN1, and LAMININ (Fig. 6b). Although collagen deposition did not significantly change following *Fermt2* and *Tns1* knockdown, as evidenced by Masson staining, FN1 was significantly decreased (Fig. 6c&d). Since laminins were enriched in the basement membrane, we focused on FN1.

Major FN1 receptors included CD44, SDC1, SDC4, and integrins (Fig. 6e), with communication probabilities differing across CMS subtypes (Fig. 6f). FN1 was significantly decreased in co-cultures following *Fermt2* or *Tns1* knockdown in L929 cells (Fig. 6g), and *Tns1* knockdown in MEFs validated these results (Fig. S6b). Moreover, FERMT2 and TNS1 protein levels significantly correlated with FN1 protein levels in patients with CRC (Fig. 6h). Phosphorylation of TNS1 (Ser1400, Ser445) and FERMT2 (Ser177) was significantly correlated with FN1 protein levels (Fig. 6i). Thus, our findings indicate that CPLF top-weighted genes regulate FN1 expression in myofibroblasts, potentially enhancing cancer cell growth.

### TNS1 and FERMT2 activate integrin signaling in cancer cells

**Fig. 7:**
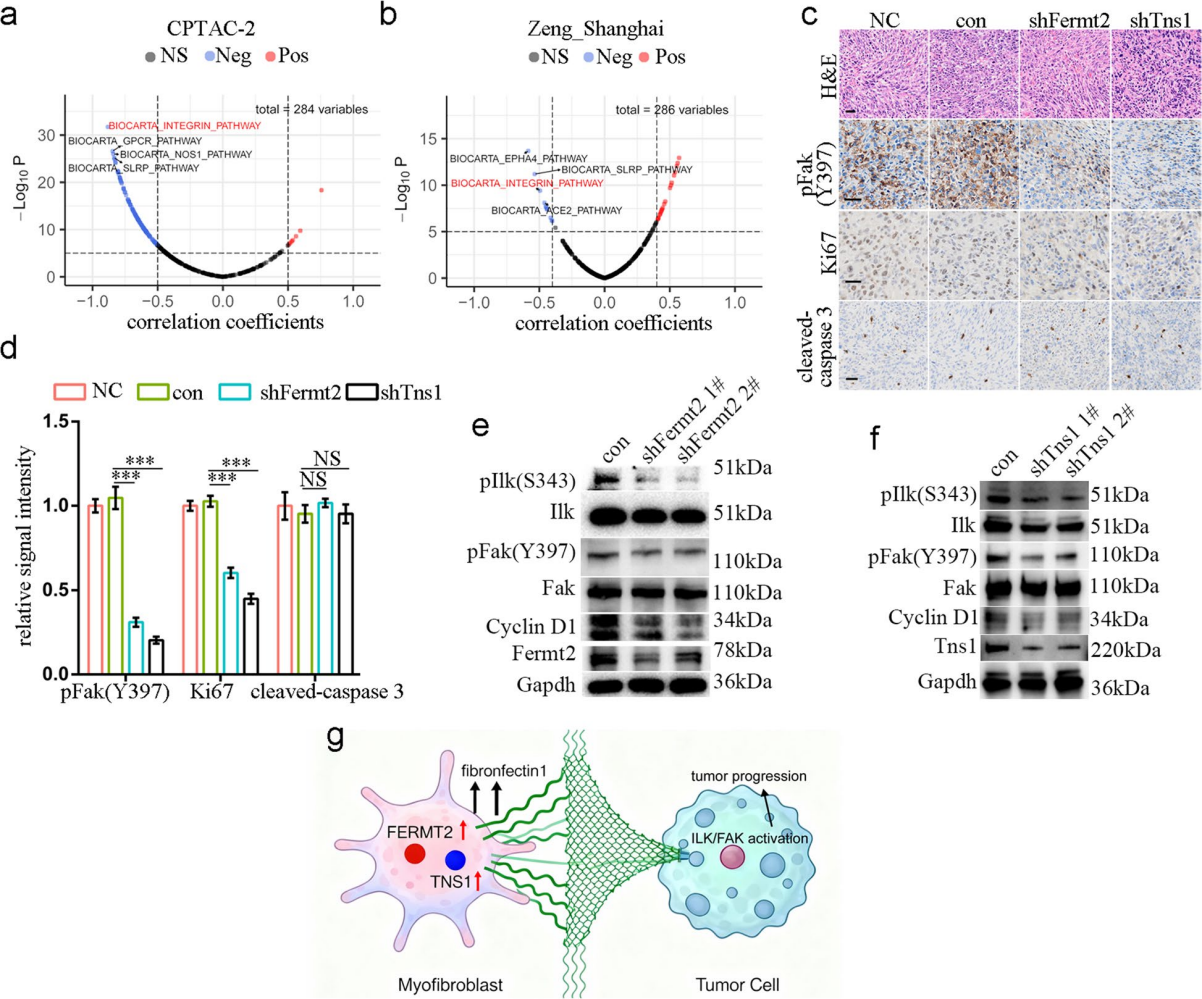
Fibronecin in fibroblast activated focal adhesion signaling in cancer cells. **a**&**b** Volcanoplot showing correlation of FN1 expression and major signaling pathways in tumor tissues from CPTAC-2 cohort (**a**) and Zeng_Shanghai cohort (**b**). **c** Representative images of staining intensities of molecules stained in subcutaneous tumor models of MC38 and L929 co-culture. scale, 100 μm. **d** Barplot showing quantitative result of signal intensity in C, n = 3 for biological replicates. ***, *P* < 0.001; NS, not significant. **e**&**f** WB result showing signal intensities of indicated molecules in in vitro co-culture model of MC38 cells with shFermt2 L929 cells (**e**) and shTns1 L929 cells (**f**). **g** A proposed model for FERMT2- and TNS1-mediated tumor progression

To investigate how TNS1 and FERMT2 expression in myofibroblast promotes cancer progression, we quantified the activities of 284 signaling pathways in CPTAC-2 proteomic data (Table S5). Correlation analysis revealed significant inverse correlation of CPLF with the integrin (*r* = − 0.882, *P* < 0.001) and small leucine-rich proteoglycan (SLRP; *r* = − 0.845, *P* < 0.001) pathways (Fig. 7a). Additionally, we quantified the activities of 284 signaling pathways in the Zeng_Shanghai cohort (Table S6). Pearson correlation analysis validated the top-ranked inverse correlation between the CPLF score and the integrin (*r* = − 0.495, *P* < 0.001) and SLRP (*r* = − 0.536, *P* < 0.001) pathways (Fig. 7b). Immunohistochemistry on tumours from mouse models validated this finding, showing significant downregulation of pFAK(397) (Fig. 7c&d), a key signaling mediator of integrin activation and cancer cell survival, upon *Tns1* and *Fermt2* inhibition. Furthermore, *Tns1* and *Fermt2* knockdown inhibited cancer cell proliferation but not apoptosis, as indicated by Ki67 and cleaved-caspase 3 staining, respectively (Fig. 7c&d).

In co-culture experiments, our model clearly demonstrated that *Fermt2* and *Tns1* inhibition in fibroblasts led to reduced phosphorylation of ILK (Ser343) and pFAK(397) (Fig. 7e&f, respectively). Moreover, this led to decreased cyclin D1 levels in cancer cells (Fig. 7e&f). Similar reductions were observed in pIlk(S343) and pFak(Y397) with *Tns1* knockdown in MEFs following co-culture with MC38 cells (Fig. S6c).

Immunofluorescence analysis confirmed reduced pIlk(S343) in cancer cells co-cultured with either *Tns1*- or *Fermt2*-knockdown L929 cells (Fig. S6d&e), although suitable antibodies for detecting pFAK(Y397) were unavailable. A concurrent reduction in pIlk(S343) was also observed in the knockdown L929 fibroblasts themselves (Fig. S6d&e). Based on our findings, we conclude that myofibroblast-derived CRC, TNS1, and FERMT2 orchestrate tumour progression by upregulating FN1, which subsequently activates integrin and FAK signaling in cancer cells (Fig. 7g).

## Discussion

The discovery of intrinsic prognostic determinants in CRC has faced several challenges, primarily due to the complexity of the molecular types (DNA, RNA, protein, and metabolites). In this study, we analysed four large-scale multi-omics datasets: CPTAC-1, CPTAC-2, Zeng-Shanghai, and Sidra-LUMC. Using MOFA +, which integrates these heterogenous omics layers to reduce data dimension and identifies shared latent factors, we uncovered patient-intrinsic molecular signatures. Among these, the discovery of a prognosis-related factor was clinically valuable, and this factor was consistently validated in all four datasets. These consistent effects across independent cohorts underscore the robustness of the latent factors identified in our multi-omics analysis.

MDF2 shows strong potential for clinical prognostication. However, unlike mutation status, which is typically binary, translating the continuous MDF2 score into a clear risk classification is not straightforward. Before MDF2 can be applied in clinical prediction, two fundamental requirements must be met: (1) establishing reference ranges to define low-risk and high-risk thresholds for the MDF2 score, and (2) developing a standardized analytical pipeline for processing RNA-seq or proteomics data from tumour samples. With these in place, MDF2 scoring can be standardized using our model weights, and the resulting value could be directly interpreted as low- or high-risk based on the established reference. Although the prognostic effect in the CPTAC-2 discovery cohort did not reach statistical significance, this likely reflects limited follow-up and sample size rather than biological irrelevance, as suggested by the successful cross-cohort replication.

ECM remodelling, a key cellular process underlying CRC progression, was enriched in CPLF [17]. The ECM comprises approximately 300 unique molecules classified into collagens, proteoglycans (hyaluronan, chondroitin sulphate proteoglycans), and glycoproteins (fibronectin, laminins) [18]. Hyaluronan [19], tenascin [20], and certain collagens [21] are frequently upregulated in CRC tissues. Although some tumour cells can secrete certain matrisome proteins (such as hyaluronic acid), fibroblasts produce the majority of the tumour ECM [22]. Fibroblasts in CRC are heterogenous and can be broadly categorized into immune-related fibroblasts and myofibroblasts, reflecting immune reaction and wound healing, respectively [23]. High-quality single-cell data showed that CPLF activity was predominantly localized to myofibroblasts. While myofibroblasts were present in both normal and tumour tissues, they showed distinct localization and functional patterns [24]. Notably, the spatial redistribution of FERMT2-positive myofibroblasts from the basal lamina in normal tissue to desmoplastic regions in tumours suggests tumour microenvironment–driven reprogramming of stromal signaling niches. Our findings indicate that myofibroblast dysfunction drives CRC progression, and this conclusion is supported by previous studies. However, the detailed mechanism underlying this phenomenon remains under debate.

Fibronectin is overexpressed in multiple cancer types, including CRC, and can predict prognosis [25]. Mechanistically, fibroblast-secreted fibronectin is recognized by integrins—primarily α5β1—on cancer cells [26]. This interaction leads to structural changes in FN1, followed by fibrillogenesis, which initiates ECM formation [26]. The FN1 fibrillar not only provides binding sites for other ECM components, such as collagen, but also for cancer cells and other growth factors, particularly TGF-β, which initiates epithelial-mesenchymal transition [27]. Our data revealed that *TNS1* and *FERMT2*, two top-weighted genes in CPLF, regulate FN1 in fibroblasts. TNS1 and FERMT2 are the structural components and signaling mediators of focal adhesion [28, 29]; however, their influence on FN1 production in fibroblasts has not been previously described. The strong positive correlations between mRNA, protein, and phosphoprotein levels for TNS1 and FERMT2, together with their top CPLF loadings, suggest coordinated regulation across omics layers that supports their central role in ECM remodelling. We postulate that fibroblast–ECM adhesion is essential for FN1 secretion.

Although TNS1 and FERMT2 are co-expressed in CRC tissues and both regulate FN1 expression in myofibroblasts, their knockdown induced distinct phenotypic and molecular changes. Both proteins are known regulators of integrin signaling, albeit through different mechanisms. FERMT2 acts as a scaffold protein that directly binds phosphatidylinositol and integrins to regulate cell morphology and signaling. Its knockout has been shown to destabilize the cell cortex and alter FAK activation in human podocytes. Additionally, FERMT2 interacts with β-catenin and TCF4 to stabilize β-catenin and promote transcriptional activation. In contrast, TNS1, while also a scaffold protein, specifically regulates the early formation of focal adhesion via its interaction with β-integrin. This is consistent with our observation that *Fermt2* knockdown induced a more rounded cell morphology in L929 cells compared to *TNS1* knockdown, while uniquely affecting YAP signaling. Furthermore, the reduction of α-SMA following *TNS1* knockdown aligns with the findings of Bernau et al. [15], who identified TNS1 as a key regulator of myofibroblast differentiation. Together, these findings suggest that post-translational modification of TNS1, particularly phosphorylation at Ser445 and Ser1400, may enhance its prognostic relevance compared to mRNA expression alone.

Unbiased pathway analysis revealed a strong correlation between CPLF and integrin/SLRP signaling. This in silico result was validated by our in vitro co-culture model, demonstrating that fibroblasts regulate ILK and FAK signaling in cancer cells. Thus, our findings have uncovered the fibroblast–tumour cell crosstalk, mediated through the FN1/integrin/tyrosine kinase pathway. This crosstalk may underpin the pro-tumorigenic role of fibroblasts. Further mechanistic studies are needed to verify this conclusion. Importantly, reduced phosphorylation of FAK(Y397) and ILK(S343) in cancer cells following Tns1 or Fermt2 suppression supports a model where fibroblast-derived signals are required to activate integrin-dependent tumour progression pathways.

However, several limitations of this study should be acknowledged. The prognostic value of CPLF was not assessed in an in-house cohort, potentially limiting its immediate real-world applicability. Moreover, the scope of our study did not include investigating how CPLF influences other tumour microenvironmental components in CRC, such as immune or stromal populations, thereby restricting a more holistic understanding of its role in tumour progression. Additionally, several insights rely on computational analyses and require experimental confirmation.

In conclusion, our study addresses a critical gap in CRC literature by identifying CPLF as a prognostic latent factor derived from multi-omics integration. We elucidate a novel mechanism whereby CPLF orchestrates myofibroblast–cancer cell crosstalk via the FN1/ILK/FAK pathway to drive tumour progression. This work not only positions CPLF as a prognostic biomarker but also highlights its pathway as a promising target for innovative stromal-directed therapies.

## Materials and methods

### Data acquisition

CPTAC-2 dataset (110 samples) was used to construct MOFA model, and was download from Cbioportal (https://www.cbioportal.org). CPTAC-1 dataset (109 samples), Sidra-LUMC dataset (348 samples) and clinical data of Zeng_Shanghai were also down loaded from Cbioportal. Proteomics and phospho-proteomics data of Zeng_Shanghai (146 patients) were downloaded from supplementary data of the paper. Single-cell RNA sequencing datasets (GSE178341 and GSE144735) were downloaded from GEO repository. Spatial data were from http://www.cancerdiversity.asia/scCRLM/.

### Data integration and model construction

Mutation, miRNA, mRNA, proteomics, phospho-proteomics from CPTAC-2 dataset were used to construct the MOFA model. All omics data except mutation data were z-score normalized. For each omic data type: genes with more than 10 mutation were included (n = 379); 100 most variable (standard deviation value) miRNAs (~ 10% of all miRNAs); 1000 most variable mRNAs; Protein (*n* = 5658) and phospho-proteins (*n* = 5519) with > 80% detection rate were included to train the model. 96 patients with all 5 omics data types available were included. Factor analysis was performed according to the MOFA tutorial with the default settings. Parameter “Factor” was set to 15, convergence_mode was set to “slow”. Basic visualization was achieved with MOFA package (version 1.14.0). For the model constructed without pre-filtering, we incorporated the aforementioned 379 mutations, 5,658 proteins, 5,519 phospho-proteins, as well as all 989 miRNAs and 13,481 mRNAs. All model parameters remained unchanged from the previous model.

### Factor 2 score calculation in validation datasets

Factor 2 in the validation datasets were calculated by the following matrix operation:

Z = YW^−1^;

Z represents factor 2 score, Y represents z-scored proteomics data, W represents factor 2 weights in our MOFA model. The resulted factor 2 score was used to downstream analysis. Significance of multi-group median differences was performed using the non-parameter statistical method Kruskal–Wallis test.

### Multivariable COX proportional hazards regression

The Cox proportional hazards regression analysis was performed using the *coxph* function from the R package "survival". The survival outcome was constructed as a Surv object, and the model included the following covariates: age, sex, metastatic site, histology, CMS type, TNM stage, and 15 additional factors.

### Enrichment analysis

Proteomic data of factor weights were first varimax transformed, followed by selection of the top 150 weighted genes. Pathway enrichment analysis were performed by using R package “clusterProfiler” and visualized using R package “enrichplot”.

### Single-cell processing

The count matrix was downloaded from the Gene Expression Omnibus. Data were processed using the standard Seurat (v5.1.0) pipeline, which included dimensionality reduction via UMAP. A gene module score for Factor 2 was computed with the AddModuleScore function, based on genes with weights greater than 0.5 identified from the proteomic data. Specifically, the full list of gene weights was subjected to varimax rotation, after which genes with weights above 0.5 were retained. The resulting gene set was further filtered by requiring a protein–mRNA expression correlation greater than 0.5, yielding a final signature of 143 genes. Unless otherwise stated, the term “MDF2 gene signature” refers to this 143-gene set. To identify the fibroblast subtype exhibiting the strongest MDF2 characteristics, we derived a more focused 11-gene signature from the original set by applying a stricter weight threshold of > 0.74. Cell types were annotated according to the metadata provided in the original publication. Spatial transcriptomic data, obtained from http://www.cancerdiversity.asia/scCRLM/, were also analyzed using Seurat. The analysis codes are publicly available at 10.6084/m9.figshare.30588521 to facilitate reproducibility of our results.

### Cell lines and antibodies

MC38 (SCSP-5431), L929 (SCSP-5039), C57BL/6 MEF (SCSP-102) and CMT-93 (TCM43) cells were from National Collection of Authenticated Cell Cultures, Chinese Academy of Sciences. All cell lines were screened for Mycoplasma contamination and authenticated using short tandem repeat (STR) profiling. All cells were cultured in DMEM with 10% fetal bovine serum and supplemented with 1 × Penicillin–Streptomycin-Gentamicin Solution (Biosharp, BL141A). All cells were maintained in a humidified incubator with 5% CO_2_ at 37 °C. Rabbit anti-FERMT2 (11,453–1-AP), anti-Tns1 (20,054–1-AP), anti-COL1A1 (67,288–1-Ig), anti-α-SMA (14,395–1-AP), anti-fibronectin (15,613–1-AP), anti-GAPDH (60,004–1-Ig), anti-FAK (12,636–1-AP), anti-ILK (12,955–1-AP) were from Proteintech (Wuhan, China). Anti-Cyclin D1 (SA748) and anti-caspase 3 (SA826) were from Sanjing Biotech (Hangzhou, China). pILK(S343) (AF8070), pFAK(Y397) (AF3398) were from Affinity Biotech (Liyang, China).

### Western blot and immunohistochemistry staining

The WB procedure and IHC protocol were indicated previously [30] with minor modificaions. Briefly, tris–glycine gels were from Bioshap with catalogue number of BL1008A, running buffer was from beyotime with catalogue number of P0014A-50bgs. Secondary antibodies were from proteintech (Cat No. SA-00001–2). For IHC staining, scanning of the images were accomplished with Olympus VS200.

### Immunofluorescence

Tumor cells and fibroblast cells were co-cultured at a density of 1 × 10^4^ cells each per well in a 24-well plate (CWBIO, CW9903L) containing glass coverslips (Thermo Fisher Scientific, 174,950) at the bottom. After 48 h of culture, cells on the coverslips were fixed with 4% formaldehyde, permeabilized with 0.2% Triton X-100, and blocked with 5% bovine serum albumin (BSA; Beyotime, ST023). Primary antibodies were diluted at 1:100 unless otherwise specified and incubated with the cells overnight at 4 °C. The following secondary antibodies from Thermo Fisher Scientific were used: Alexa Fluor 555-conjugated goat anti-Rabbit IgG (A-21428) and Alexa Fluor 488-conjugated donkey anti-Rabbit IgG (A-21206). Images were acquired using a confocal microscope (LSM 880 NLO FILM; Zeiss, Cambridge, UK).

### Multiplex IHC staining and imaging

Tissue sections underwent deparaffinization followed by treatment with antigen unmasking solution (ServiceBio, G1202). 30 min incubation with 10% goat serum to block unspecific binding were performed before incubating with primary antibodies overnight at 4 °C. Sections were washed with PBST for 3 times followed by incubation with SignalStain® Boost IHC detection Reagent (Cell Signaling Technology, 8059) for 30 min in a moist atmosphere in darkroom. Tyramide signal amplification staining (1:100) was used label positive signal. The second round was started with antigen retrieval. Finally, 4′,6-diamidino-2-phenylindole (DAPI; ServiceBio, G1012) was employed to visualize the nuclei within the cells. VS200 microscope (Olympus) was used to image sections and data were analyzed by the respective software.

### Stable cell line construction

Cell transfection was performed using Calcium Phosphate Cell Transfection Kit (Beyotime, C0508). Viruses were generated by co-transfecting HEK293T cells with psPAX2, pMD2.G, and PLKO.1 or shFermt2 or shTns1. The virus particles were then collected for infection of L929 cells. Cells were selected with appropriate concentrations of puromycin (Beyotime, ST551).

### Cell growth assay

Cell growth was determined using Cell Counting Kit-8 (CCK8) assays (FD3788, Fude Biotech, Hangzhou, China). Cell viability was assessed after a 2-h incubation with CCK-8 solution. The conversion of WST-8 to formazan was measured at 450 nm using a microplate detection system (Molecular System, SpectraMax iD3).

### Co-culture of cancer cells and fibroblastic cells

The detailed procedure for the co-culture system used in IF staining is provided in the respective IF section. For WB detection, MC38 cancer cells and L929 fibroblastic cells were co-cultured at a density of 1 × 10⁶ cells each in 10-cm dishes. After 48 h of co-culture, cells were immediately placed on ice, washed with ice-cold PBS, and subsequently lysed for WB analysis.

### Subcutaneous mouse model

Animal experiments were performed according to procedures approved by the Institutional Animal Care and Use Committee at the Hangzhou Medical College (Approval No. ZJCLA-IACUC-20010958). 70% confluent MC38 cells and L929 cells or MEF cells were collected and mixed at 1:1 ration in DMEM. 100 μl mixture containing 1 × 10^6^ MC38 cells and L929 cells or MEF cells were inoculated into the left flank of 8-week-old C57BL/6 mice (Shanghai SLAC Laboratory Animal Co. Ltd., Shanghai, China). All animal experiments were conducted under specific pathogen-free (SPF) conditions. Mice were housed in a controlled environment maintained at 22 ± 2 °C with 50–60% humidity, under a 12-h light/12-h dark cycle (lights on at 7:00 AM). Animals were provided with autoclaved standard rodent chow and sterile water ad libitum, and were housed in individually ventilated cages (IVCs) with corn cob bedding, which was changed weekly. Each experimental group consisted of 4 randomly assigned mice. Tumor volume was monitored every 3 days. At day 15 post injection, all mice were sacrificed and tumors were separated, washed, weighted, pictured and fixed in 4% PFA (G1101, ServiceBio). The tumor tissue bulk was further embedded into paraffin.

### Human colorectal cancer specimen

Human CRC specimens were obtained from the Department of Pathology at Hangzhou First People’s Hospital. The study utilized 5 paired samples, each set comprising CRC tissue and matched adjacent normal mucosa from the same patient. All tissues were formalin-fixed and paraffin-embedded (FFPE). The use of these specimens was approved by the Institutional Review Board (IRB) of Hangzhou First People’s Hospital (Approval No. 2025KY061-1), with a waiver of informed consent given the retrospective nature of the study.

### Section staining with Alcian blue and masson

Alcian blue staining was performed with kit (ServiceBio, GP1040) and proceeded with the manual. Masson staining was implemented with kit GP1032v of ServiceBio. Images were recorded with Olympus VS200 system.

### Statistics

The sample size for the animals was not determined using a statistical method. Instead, the selection was guided by our familiarity with the experimental models commonly employed in cell biology and animal research. All data shown were obtained from at least three biological independent experiments with similar results. Upon confirmation that the values followed a normal distribution, a two-tailed Student’s *t*-test was applied to determine the significance of differences between two independent sample groups. Pearson correlation analysis was used to determine the correlation between two variables. Comparisons of means across multiple groups were performed using the Kruskal–Wallis test. Survival rates were analyzed using COX proportion model (log-rank test). Statistical analyses were conducted using GraphPad Prism software (version 8.0). All *P* values are indicated in the figures. Significance was denoted as follows: *P < 0.05, **P < 0.01, ***P < 0.001; NS., not significant. Unless stated otherwise, all biological experiments were repeated three times.

## Supplementary Information

Supplementary Material 1.

Supplementary Material 2.

## Data Availability

The datasets analysed during the current study are available in the Cbioportal repository at https://www.cbioportal.org/. MOFA model and Codes to reproduce this study were available at 10.6084/m9.figshare.30588521.
