# Supplementary material for: Multi-omics factor analysis identifies the Tensin 1-Fermitin family homologue 2–Fibronectin 1–Integrin signaling axis as a prognostic determinant in colorectal cancer
Source: Mol Biomed. 2025 Dec 17;6:141. doi: 10.1186/s43556-025-00386-0 (PMC12711624; doi:10.1186/s43556-025-00386-0)
Supplement: Supplementary file 1 — Supplementary Material 1. [file 43556_2025_386_MOESM1_ESM.docx]

**Multi-omics Factor Analysis Identifies the Tensin 1-** **Fermitin family homologue 2–Fibronectin 1–Integrin Signaling Axis as a Prognostic Determinant in Colorectal Cancer**

**Tianwei Chen^1,2#*^, Yebin Yang^2#^, Jing Shi^3#^, Fanhe Dong^2^, Lesi Xie^4^, Yuqiang Shan^2*^, Xiang Wang^1*^**

^1^ Zhejiang Key Laboratory of Zero Magnetic Medicine, Affiliated Hangzhou First People's Hospital, School of Medicine, Westlake University, China

^2^ Department of Gastrointestinal Surgery, Affiliated Hangzhou First People's Hospital, School of Medicine, Westlake University, China

^3^ Department of General Surgery, Zhejiang Hospital, Hangzhou, Zhejiang Province, China

^4^ Department of Pathology, Affiliated Hangzhou First People's Hospital, School of Medicine, Westlake University, China

**^#^ Tianwei Chen, Yebin Yang and Jin Shi contributed equally to this work**

*** Correspondence:**

**Xiang Wang:** Zhejiang Key Laboratory of Zero Magnetic Medicine, Affiliated Hangzhou First People's Hospital, School of Medicine, Westlake University, China. [wangxiang2021@zju.edu.cn](mailto:wangxiang2021@zju.edu.cn)

**Yuqiang Shan:** Department of Gastrointestinal Surgery, Affiliated Hangzhou First People ' s Hospital, School of Medicine, Westlake University, China. shang110117@163.com.

**Tianwei Chen:** Zhejiang Key Laboratory of Zero Magnetic Medicine, Affiliated Hangzhou First People's Hospital, School of Medicine, Westlake University, China. chentianwei@sibs.ac.cn.

**Supplementary directory**

Figures and Legends ............................................................................................ **2**

Tables ................................................................................................................... **8**

**Supplementary Figures and legends**


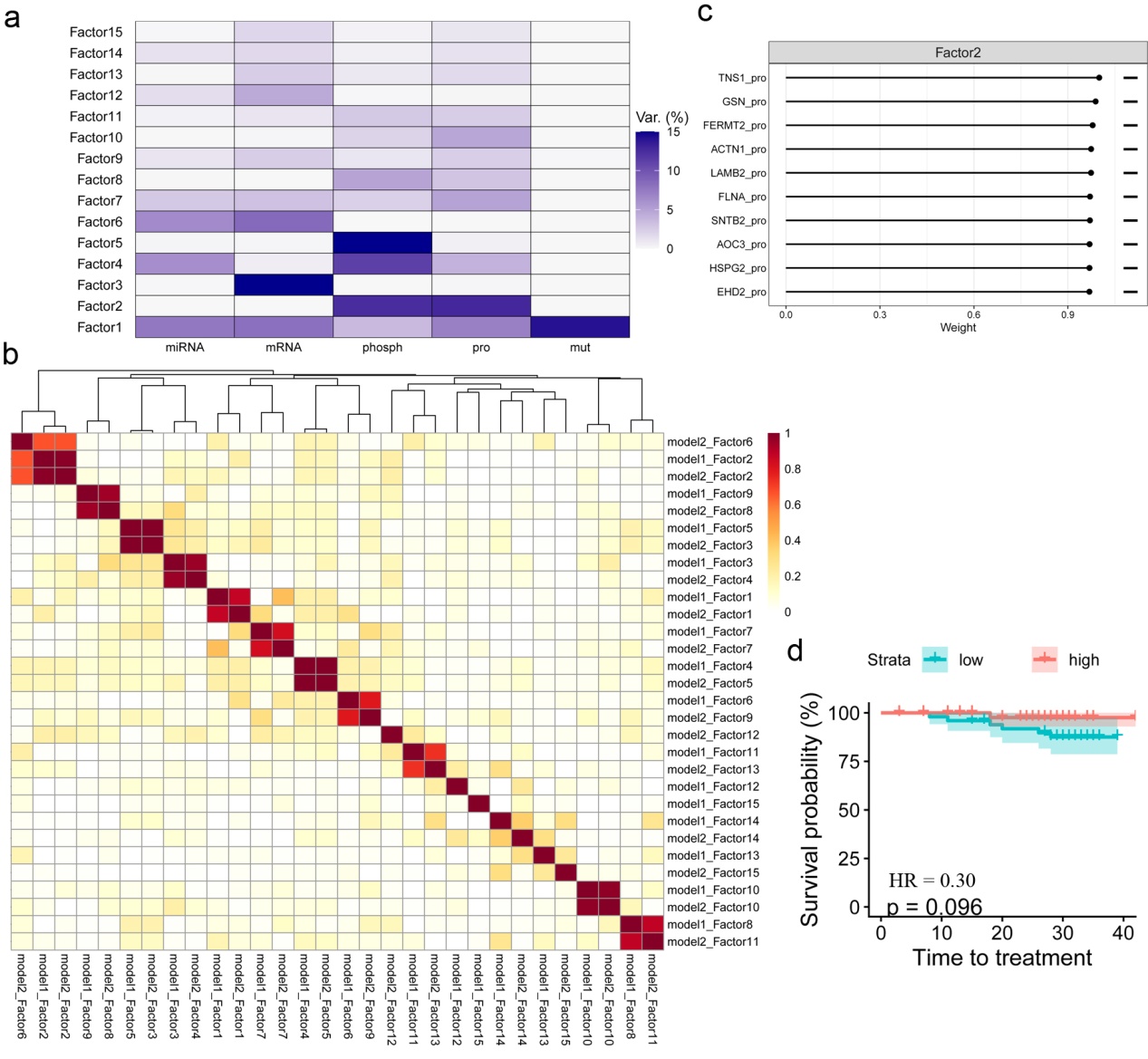


**Figure S1 Stability of MDF2 in the MOFA model without pre-filtering.** (a) Bar plot showing variance explained by different factors. (b) Heatmap showing correlations between factors from two MOFA models. Model1 represents the original model and model2 represents the new model. (c) Lollipop chart indicating feature weights of Factor 2 in the new model. (d) Kaplan-Meier plot showing correlation of survival status and Factor 2 score in cohort CPTAC-2 (samples in low group = 32, samples in high group = 64).


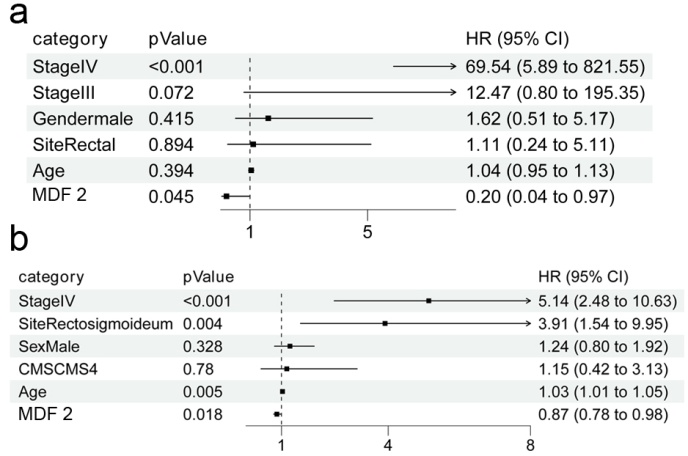


**Figure S2 MDF2 identified as an independent prognostic factor by multivariable Cox analysis.** Forest plot demonstrating the hazard ratio (HR) and statistic p value of multivariable COX regression result of Zeng_Shanghai cohort (a) and Sidra_LUMC cohort (b).


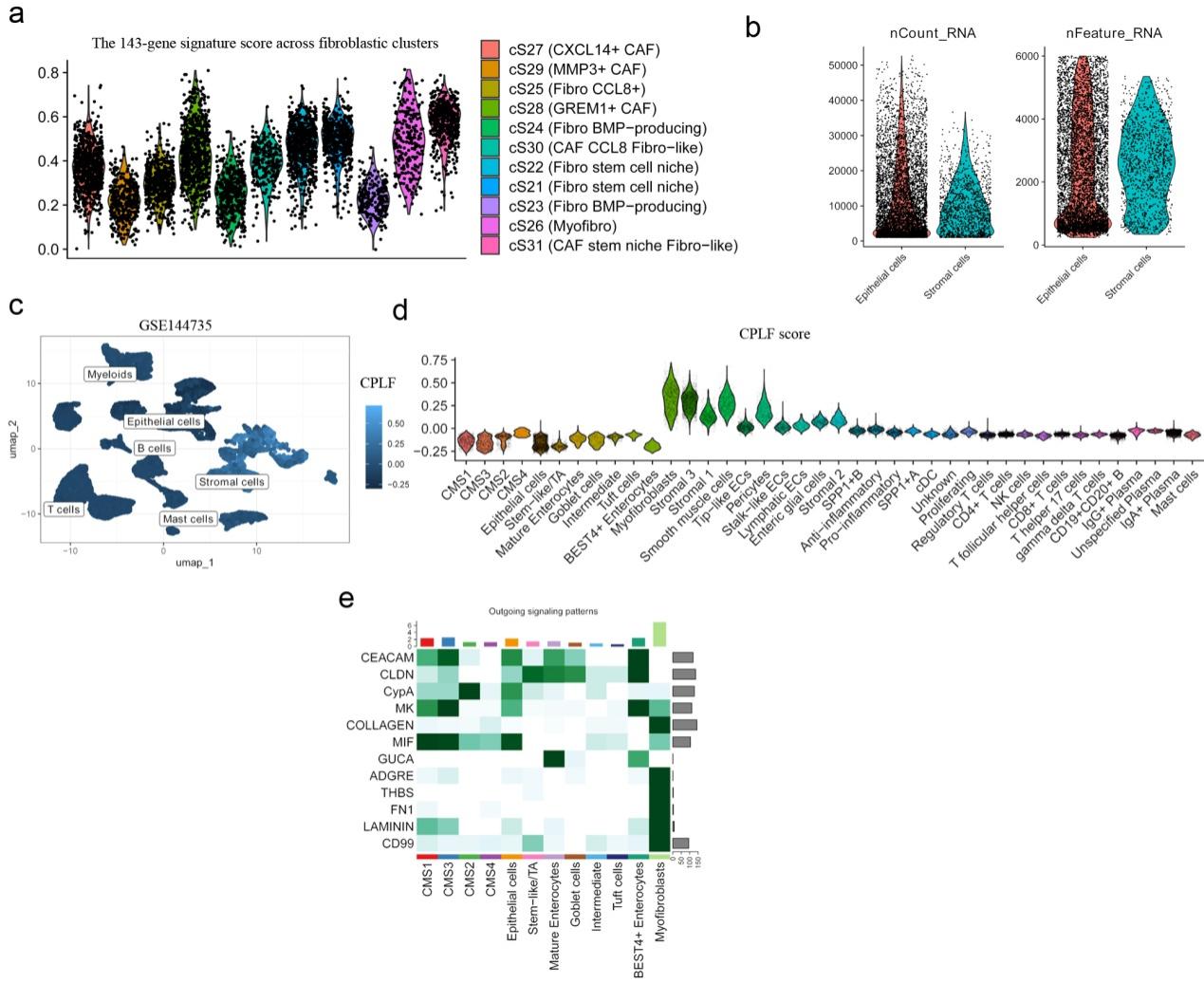


**Figure S3 CPLF mainly expressed in myofibroblasts validated in GSE14475 cohort.** (a) Violin plot showing CPLF signature score in different fibroblastic subtypes from GSE178341. (b) Violin plot showing count and feature numbers of cells in GSE 14475. (c) UMAP plot demonstrating low-dimensional localization of different cell types. (d) Violin plot showing CPLF score in different cell subtypes. (e) Heatmap showing outgoing signals of myofibroblasts and cancer cells.


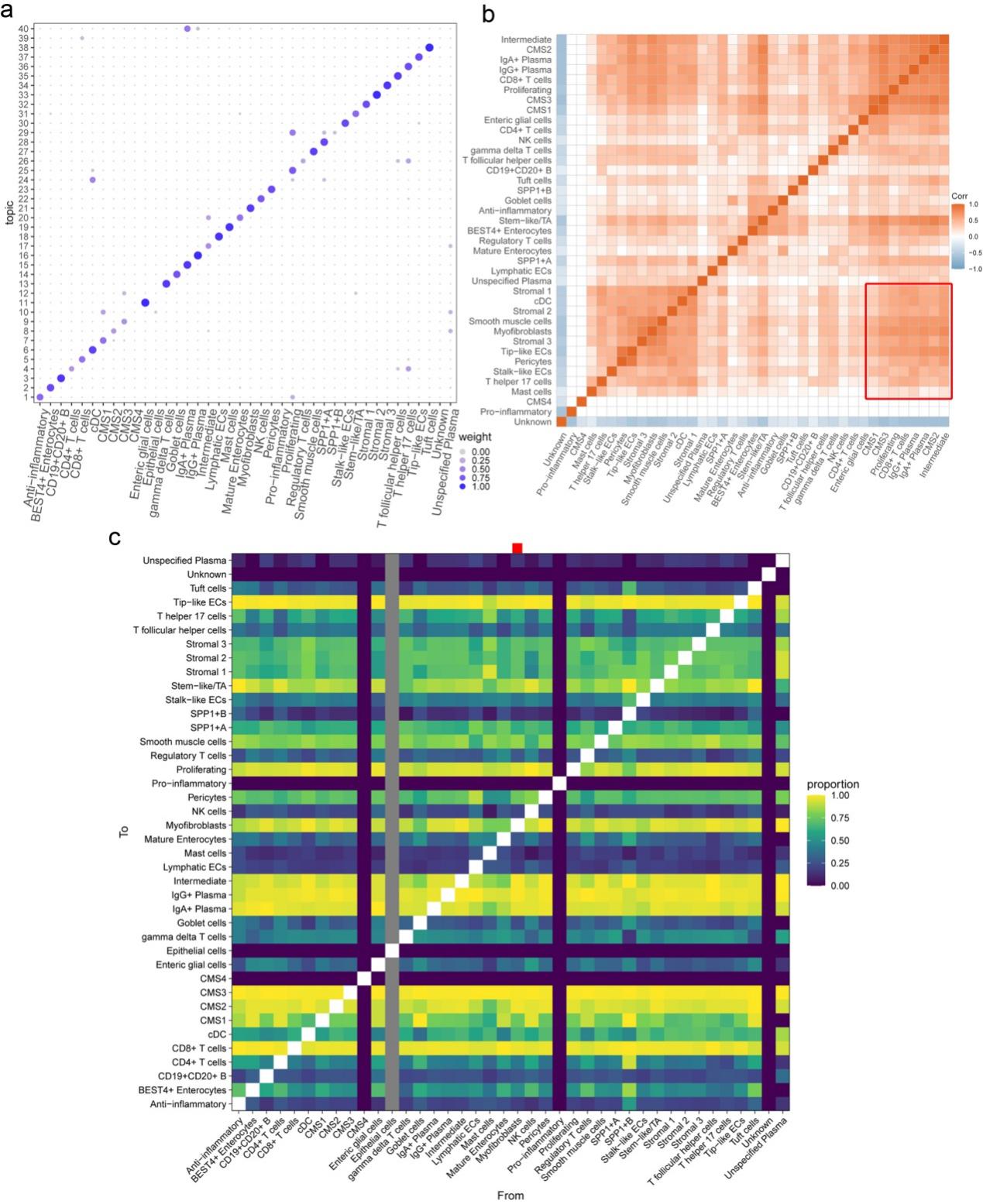


**Figure S4 Myofibroblasts are located adjacent to cancer cells.** (a) Correlation plot showing the similarity between cell types deconvoluted by Spotlight. (b) Correlation plot demonstrating clusters of cell types. (c) Heatmap showing interaction between cell types.


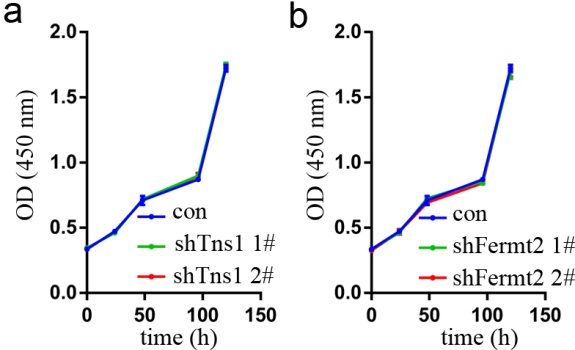


**Figure S5 Tns1 or Fermt2 knockdown does not affect cell growth.** Line plots comparing the growth rates of control and knockdown cells for shTns1 (a) and shFermt2 (b), n = 3 for biological replicates.

**
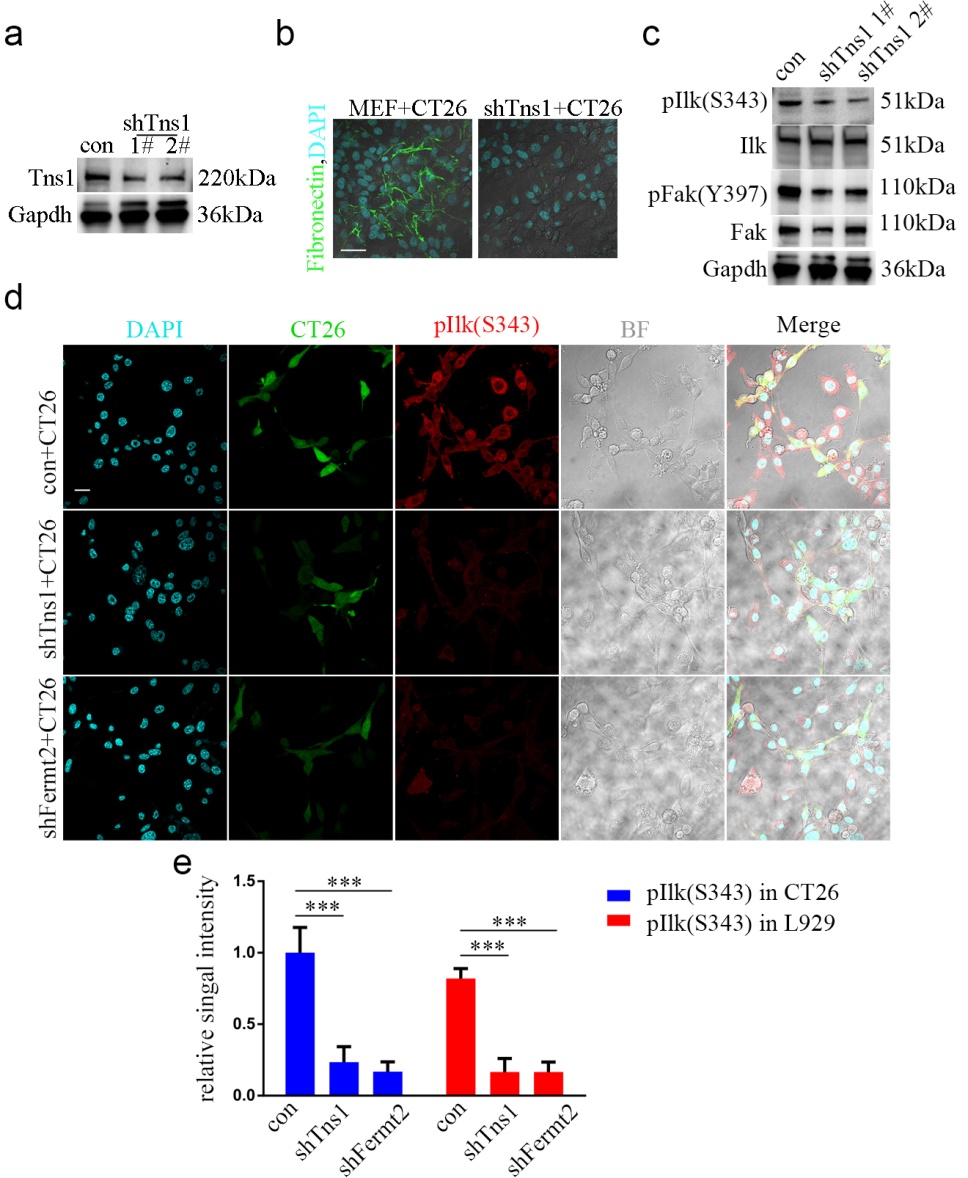
**

**Figure S6 TNS1 knockdown suppresses ILK and FAK phosphorylation.** (a) WB result showing efficacy of Tns1 KD in mouse embryonic fibroblast (MEF) cells. (b) Representative images showing result of immunofluorescence staining FN1 in different co-culture groups. Scale bar, 40 μm. (c) WB result showing expression levels of indicated molecules in different groups. (d) Representative images demonstrating signal intensities of indicated molecules. Scale bar, 40 μm. (e) Bar plot showing the comparison of signal intensities in (d), n = 5 for biological replicates. ***, P<0.001.

**Supplementary Tables**

**Table S1 Baseline clinicopathological characteristics of the CPTAC-2 cohort**

|  | **Number** | **Total** |
| --- | --- | --- |
|  | ***N=110*** |  |
| Age | 65.0 (11.9) | 108 |
| Sex: |  | 110 |
| Female | 65 (59.1%) |  |
| Male | 45 (40.9%) |  |
| Overall Survival Status: |  | 102 |
| DECEASED | 8 (7.84%) |  |
| LIVING | 94 (92.2%) |  |
| Overall Survival (Months) | 28.0 (7.26) | 102 |
| Disease Free Status: |  | 97 |
| DiseaseFree | 81 (83.5%) |  |
| Recurred/Progressed | 16 (16.5%) |  |
| Disease Free (Months) | 28.8 (6.30) | 85 |
| Histology: |  | 109 |
| Mucinous | 21 (19.3%) |  |
| Not Mucinous | 88 (80.7%) |  |
| Tumor Site: |  | 109 |
| Ascending Colon | 27 (24.8%) |  |
| Cecum | 23 (21.1%) |  |
| Descending Colon | 10 (9.17%) |  |
| Hepatix Flexure | 7 (6.42%) |  |
| Sigmoid Colon | 38 (34.9%) |  |
| Splenic Flexure | 3 (2.75%) |  |
| Tranverse Colon | 1 (0.92%) |  |
| Pathology T Stage: |  | 110 |
| T2 | 16 (14.5%) |  |
| T3 | 80 (72.7%) |  |
| T4a | 12 (10.9%) |  |
| T4b | 2 (1.82%) |  |
| Pathology N Stage: |  | 110 |
| N0 | 56 (50.9%) |  |
| N1 | 6 (5.45%) |  |
| N1a | 14 (12.7%) |  |
| N1b | 18 (16.4%) |  |
| N2a | 9 (8.18%) |  |
| N2b | 7 (6.36%) |  |
| Cancer Stage: |  | 110 |
| Stage I | 12 (10.9%) |  |
| Stage II | 42 (38.2%) |  |
| Stage III | 48 (43.6%) |  |
| Stage IV | 8 (7.27%) |  |
| History of Polyps: |  | 97 |
| No | 76 (78.4%) |  |
| Yes | 21 (21.6%) |  |
| Polyps Currently Present.: |  | 107 |
| No | 75 (70.1%) |  |
| Yes | 32 (29.9%) |  |
| MSI Status: |  | 105 |
| MSI-H | 24 (22.9%) |  |
| MSS | 81 (77.1%) |  |
| CMS classification: |  | 106 |
| CMS1 | 18(16.98%) |  |
| CMS2 | 29(27.36%) |  |
| CMS3 | 17(16.04%) |  |
| CMS4 | 33(31.13%) |  |
| Mixed | 9(8.49%) |  |

**Table S2 Baseline clinicopathological characteristics of the Zeng_Shanghai cohort**

|  | **Number** | **Total** |
| --- | --- | --- |
|  | ***N=146*** |  |
| Gender: |  | 146 |
| female | 47 (32.2%) |  |
| male | 99 (67.8%) |  |
| Age | 64.7 (7.91) | 146 |
| Tumor site: |  | 146 |
| Asending Colon | 19 (13.0%) |  |
| Asending Colon;Splenic Flexure | 1 (0.68%) |  |
| Cecum | 4 (2.74%) |  |
| Desending Colon | 3 (2.05%) |  |
| Hepatic Flexure | 12 (8.22%) |  |
| Rectosigmoid Junction | 6 (4.11%) |  |
| Rectum | 68 (46.6%) |  |
| Sigmoid Colon | 25 (17.1%) |  |
| Splenic Flexure | 6 (4.11%) |  |
| Transverse | 2 (1.37%) |  |
| Stage: |  | 146 |
| 0 | 1 (0.68%) |  |
| I | 15 (10.3%) |  |
| II | 33 (22.6%) |  |
| III | 27 (18.5%) |  |
| IV | 70 (47.9%) |  |
| Metastasis: |  | 146 |
| M | 70 (47.95%) |  |
| noM | 76 (52.05%) |  |
| Hitopathologic type: |  | 146 |
| High-grade intraepithelial neoplasia | 1 (0.68%) |  |
| Moderately differentiated adenocarcinoma | 134 (91.78%) |  |
| Poorly differentiated adenocarcinoma | 11 (7.53%) |  |
| CEA | 25.4 (78.3) | 139 |
| CA19.9 | 61.4 (167) | 128 |
| Survival: |  | 146 |
| Alive | 115 (78.8%) |  |
| Death | 29 (19.9%) |  |
| Loss | 2 (1.37%) |  |
| SurvivalTime | 943 (539) | 141 |
| Progression: |  | 144 |
| No | 88 (61.1%) |  |
| Yes | 56 (38.9%) |  |
| ProgressionTime | 862 (546) | 141 |

**Table S3 Baseline clinicopathological characteristics of the Sidra_LUMC cohort**

|  | **Number** | **Total** |
| --- | --- | --- |
|  | ***N=348*** |  |
| Sex: |  | 348 |
| Female | 166 (47.7%) |  |
| Male | 182 (52.3%) |  |
| Tumor site: |  | 348 |
| Ceceum | 79 (22.7%) |  |
| Colon Ascendens | 52 (14.9%) |  |
| Colon Descendens | 14 (4.02%) |  |
| Colon Sigmoideum | 121 (34.8%) |  |
| Colon Transversum | 25 (7.18%) |  |
| Flexura Hepatica | 27 (7.76%) |  |
| Flexura Lienalis | 20 (5.75%) |  |
| Rectosigmoideum | 10 (2.87%) |  |
| Tumor histology: |  | 348 |
| Adenocarcinoma | 198 (56.9%) |  |
| Adenocarcinoma In Villeus Adenoma | 2 (0.57%) |  |
| Adenocarcinoma Intestinal Type | 76 (21.8%) |  |
| Adenocarcinoma With Mixed Subtypes | 3 (0.86%) |  |
| Cribriform Carcinoma | 2 (0.57%) |  |
| Mucineus Adenocarcinoma | 64 (18.4%) |  |
| Signet Ring Cell Carcinoma | 3 (0.86%) |  |
| TNM stage: |  | 348 |
| I | 55 (15.8%) |  |
| II | 122 (35.1%) |  |
| III | 110 (31.6%) |  |
| IV | 61 (17.5%) |  |
| Age | 68.2 (11.5) | 348 |
| Survival status: |  | 348 |
| Alive | 192 (55.2%) |  |
| Dead | 156 (44.8%) |  |
| Survival time (months) | 64.2 (45.7) | 348 |
| PFS status: |  | 348 |
| DiseaseFree | 239 (68.7%) |  |
| Recurred | 109 (31.3%) |  |
| PFS time (months) | 57.9 (48.9) | 348 |
| CMS: |  | 348 |
| CMS1 | 43 (12.4%) |  |
| CMS2 | 76 (21.8%) |  |
| CMS3 | 66 (19.0%) |  |
| CMS4 | 82 (23.6%) |  |
| mixed | 81 (23.3%) |  |

**Table S4 Baseline clinicopathological characteristics of the CPTAC-1 cohort**

|  | **Number** | **Total** |
| --- | --- | --- |
|  | ***N=85*** |  |
| Gender: |  | 85 |
| female | 38 (44.7%) |  |
| male | 47 (55.3%) |  |
| Age | 69.1 (12.3) | 85 |
| Primary site: |  | 85 |
| Colon | 57 (67.1%) |  |
| Rectosigmoid junction | 7 (8.24%) |  |
| Rectum | 21 (24.7%) |  |
| MMR protein loss: NO | 84 (100%) | 84 |
| Lymphatic invasion: |  | 77 |
| NO | 36 (46.8%) |  |
| YES | 41 (53.2%) |  |
| Survival status: |  | 85 |
| Alive | 66 (77.6%) |  |
| Dead | 19 (22.4%) |  |
| Survival time (days) | 627 (616) | 77 |
| Tumor site: |  | 85 |
| Ascending colon | 11 (12.9%) |  |
| Cecum | 13 (15.3%) |  |
| Colon, NOS | 8 (9.41%) |  |
| Descending colon | 1 (1.18%) |  |
| Hepatic flexure of colon | 1 (1.18%) |  |
| Rectosigmoid junction | 7 (8.24%) |  |
| Rectum, NOS | 21 (24.7%) |  |
| Sigmoid colon | 20 (23.5%) |  |
| Transverse colon | 3 (3.53%) |  |
| TNM stage: |  | 85 |
| stage i | 15 (17.6%) |  |
| stage ii | 13 (15.3%) |  |
| stage iia | 21 (24.7%) |  |
| stage iib | 2 (2.35%) |  |
| stage iii | 10 (11.8%) |  |
| stage iiib | 7 (8.24%) |  |
| stage iiic | 8 (9.41%) |  |
| stage iv | 9 (10.6%) |  |
| Disease type: |  | 85 |
| Adenomas and Adenocarcinomas | 73 (85.9%) |  |
| Complex Epithelial Neoplasms | 1 (1.18%) |  |
| Cystic, Mucinous and Serous Neoplasms | 11 (12.9%) |  |

**Table S5 Deconvoluted pathway activity in CPTAC-2 cohort tumors**

Table S5 was available in the website in excel format.

**Table S6 Deconvoluted pathway activity in Zeng_Shanghai cohort tumors**

Table S5 was available in the website in excel format.
